# Glossology, or the Additional Means of Diagnosis of Disease to Be Derived from Indications and Appearances of the Tongue

**Published:** 1844-04

**Authors:** 


ART. II.—*Glossology, or the additional means of Diagnosis of Disease to be derived from Indications and Appearances of the Tongue.* By BENJAMIN RIDGE, M.D.—*London,* 1844. 8vo, pp. 84. With Plates.

DR. BENJAMIN RIDGE asserts in this volume that certain parts of the tongue are so connected, anatomically and physiologically, with the various viscera, and are so influenced by the affections of those viscera, that the diagnosis of individual disease can be learned from its inspection alone. Mapping out the tongue, like a phrenological bust, he asserts that the sides belong to the kidneys, the tip to the large intestines, the edges to the brain, &c.; and dividing its surface into a dozen smaller compartments, he tells us that one of these little squares belongs to the larynx and trachea, another to the bronchi, a third to the minuter bronchial tubes, a fourth to the pharynx and œsophagus, a fifth to the stomach, and so on.

The author of these "novel disclosures" (as he terms them) discourses magniloquently on his truths being buffeted, beaten down, and trampled under foot for the time by his opponents, and consoles himself with the lofty stoicism that "it is satisfactory to think a future generation will sit in judgment between them and me." The "novel disclosures" are proved, not by cases, not by post-mortem examinations, but by bare assertion that such a connexion does exist. For instance:

"The short-piled gray, or slate-coloured velvety tongue, is connected with a state of the system most difficult to manage. It is diagnostic of the firm hold which the close morbid deposit has upon the surface of every mucous membrane throughout the body."

A close morbid deposit firmly adherent to the whole gastro-intestinal mucous membrane, to the lining of the mouth, ears, nares, larynx, trachea, and bronchial membrane, to the conjunctiva, urethra, bladder—and indicated by a slate-coloured tongue! And this is the evidence on which we are called upon to receive these new revelations. It need not be said that such statements afford no satisfaction to any reader acquainted with the common rules for estimating the value of medical evidence. We here meet with assertions such as would not be justified by the largest experience. Morbid phenomena are explained as boldly and undoubtingly as if the internal processes could be actually seen; and then Dr. B. Ridge tells his readers that his observations have been made in a limited circle during the few years he has been in practice. (p. 75.) The mode in which he conducts his observations very clearly indicates that faulty method of mind which renders them valueless:

"It is my practice, in most cases, not to allow a patient to tell me his sick tale, till I have seen his tongue. When my observations and diagnosis are completed, I then say what is my opinion of his malady, requesting him to be candid with me, and state how far it agrees with what he has to complain of; should there be any difference, this leads me to examine the tongue more minutely. Something, probably, that might have been overlooked at first, is made apparent; or should this not be the case, and I see no reason to alter my first diagnosis, *I do not hesitate to differ from him; at all events, I am not misled by anything he has to say.*" (p. 52.)

We have quoted this at length, as it is a glaring and instructive example of a *petitio principii*—taking for granted what is to be proved. Dr. Benjamin Ridge so firmly believes that his scheme is true, that he absolutely rejects what contradicts it.

Again—"One circumstance," says the author, with exquisite naïveté, "has troubled me in my researches, that I have not been able to find a place on the tongue for diseases of the uterus or the male genitals." Nature, alas, as she is apt to do, contradicts the theorist, and "troubles" him. He is forced to admit that the uterus "does play a prominent part" in the diseases of women, but how is it that there is no little square uterine patch left upon the tongue? "There must, I am sure, be some reason why this has escaped me, though I believe that organ to be affected very much through the imperfect or morbid action of other organs." He feels half-conscious that this unfortunate uterus spoils the uniformity and completeness of a scheme otherwise *totus teres atque rotundus;* and he partially comforts himself with the belief that, after all, the uterus is not so very important an organ; that it is not so often the primary seat of disease.

Dr. B. Ridge has evidently industry and zeal; but the predominating faculty of his mind seems to be a scheming fancy, which leads him with fatal facility to frame theories and to discover causes, and which disinclines him to subject these "novel disclosures" to the severe and painful scrutiny of the understanding—which scrutiny, however, supposes a training of the reasoning powers, to which such a mind has never submitted itself.

Taking those things for granted which are to be proved—putting assertions in the place of arguments—allowing the wishes to warp the belief—supposing a greater uniformity and order and completeness in things than ever can be found—are defects of so grave a kind in the mind of an inquirer, that no advancement in the discovery of truth can be hoped for until they are eradicated. The existence of such defects renders nugatory the assertions, statements, inferences, and disclosures of any writer in whom these deficiencies are conspicuous, who brings forward new views. The only use of such books as this is perhaps to the writer of them, who, by publishing to the world his own mental deficiencies, gets a chance of being informed of them in an equally public way; a dear experience in every sense, but not the less beneficial because somewhat unpalatable.

